# Consensus‐Based Technical Recommendations for Clinical Translation of Renal Phase Contrast MRI

**DOI:** 10.1002/jmri.27419

**Published:** 2020-11-02

**Authors:** Anneloes de Boer, Giulia Villa, Octavia Bane, Michael Bock, Eleanor F. Cox, Ilona A. Dekkers, Per Eckerbom, Maria A. Fernández‐Seara, Susan T. Francis, Bryan Haddock, Michael E. Hall, Pauline Hall Barrientos, Ingo Hermann, Paul D. Hockings, Hildo J. Lamb, Christoffer Laustsen, Ruth P. Lim, David M. Morris, Steffen Ringgaard, Suraj D. Serai, Kanishka Sharma, Steven Sourbron, Yasuo Takehara, Andrew L. Wentland, Marcos Wolf, Frank G. Zöllner, Fabio Nery, Anna Caroli

**Affiliations:** ^1^ Department of Radiology University Medical Center Utrecht, Utrecht University Utrecht The Netherlands; ^2^ Department of Bioengineering Istituto di Ricerche Farmacologiche Mario Negri IRCCS Bergamo Italy; ^3^ Biomedical Engineering and Imaging Institute/Radiology Icahn School of Medicine at Mount Sinai New York New York USA; ^4^ Department of Radiology ‐ Medical Physics, Medical Center University of Freiburg, Faculty of Medicine, University of Freiburg Freiburg Germany; ^5^ Sir Peter Mansfield Imaging Centre, School of Physics and Astronomy University of Nottingham Nottingham UK; ^6^ Department of Radiology Leiden University Medical Center Leiden The Netherlands; ^7^ Department of Surgical Sciences Uppsala University Uppsala Sweden; ^8^ Department of Radiology Clínica Universidad de Navarra Pamplona Spain; ^9^ Department of Clinical Physiology, Nuclear Medicine and PET, Rigshospitalet Copenhagen University Hospital Copenhagen Denmark; ^10^ Department of Medicine University of Mississippi Medical Center Jackson Mississippi USA; ^11^ Department of Clinical Physics and Bioengineering NHS Greater Glasgow and Clyde Glasgow UK; ^12^ Computer Assisted Clinical Medicine, Mannheim Institute for Intelligent Systems in Medicine, Medical Faculty Mannheim Heidelberg University Mannheim Germany; ^13^ Antaros Medical BioVenture Hub Mölndal Sweden; ^14^ Department of Clinical Medicine, MR Research Centre Aarhus University Aarhus Denmark; ^15^ Departments of Radiology, Surgery and Medicine The University of Melbourne Parkville Victoria Australia; ^16^ Department of Radiology Austin Health Heidelberg Victoria Australia; ^17^ Centre for Inflammation Research University of Edinburgh, Edinburgh Bioquarter Edinburgh UK; ^18^ Department of Radiology Children's Hospital of Philadelphia Philadelphia Pennsylvania USA; ^19^ Department of Imaging, Infection, Immunity and Cardiovascular Disease The University of Sheffield Sheffield UK; ^20^ Department of Fundamental Development for Advanced Low Invasive Diagnostic Imaging Nagoya University, Graduate School of Medicine Nagoya Japan; ^21^ Department of Radiology Stanford University Stanford California USA; ^22^ High Field MR Center, Center for Medical Physics and Biomedical Engineering Medical University of Vienna Vienna Austria; ^23^ Developmental Imaging and Biophysics Section UCL Great Ormond Street Institute of Child Health London UK

**Keywords:** phase‐contrast MRI, kidney, renal blood flow, standardization, consensus

## Abstract

**Background:**

Phase‐contrast (PC) MRI is a feasible and valid noninvasive technique to measure renal artery blood flow, showing potential to support diagnosis and monitoring of renal diseases. However, the variability in measured renal blood flow values across studies is large, most likely due to differences in PC‐MRI acquisition and processing. Standardized acquisition and processing protocols are therefore needed to minimize this variability and maximize the potential of renal PC‐MRI as a clinically useful tool.

**Purpose:**

To build technical recommendations for the acquisition, processing, and analysis of renal 2D PC‐MRI data in human subjects to promote standardization of renal blood flow measurements and facilitate the comparability of results across scanners and in multicenter clinical studies.

**Study Type:**

Systematic consensus process using a modified Delphi method.

**Population:**

Not applicable.

**Sequence Field/Strength:**

Renal fast gradient echo‐based 2D PC‐MRI.

**Assessment:**

An international panel of 27 experts from Europe, the USA, Australia, and Japan with 6 (interquartile range 4–10) years of experience in 2D PC‐MRI formulated consensus statements on renal 2D PC‐MRI in two rounds of surveys. Starting from a recently published systematic review article, literature‐based and data‐driven statements regarding patient preparation, hardware, acquisition protocol, analysis steps, and data reporting were formulated.

**Statistical Tests:**

Consensus was defined as ≥75% unanimity in response, and a clear preference was defined as 60–74% agreement among the experts.

**Results:**

Among 60 statements, 57 (95%) achieved consensus after the second‐round survey, while the remaining three showed a clear preference. Consensus statements resulted in specific recommendations for subject preparation, 2D renal PC‐MRI data acquisition, processing, and reporting.

**Data Conclusion:**

These recommendations might promote a widespread adoption of renal PC‐MRI, and may help foster the set‐up of multicenter studies aimed at defining reference values and building larger and more definitive evidence, and will facilitate clinical translation of PC‐MRI.

**Level of Evidence:**

1

**Technical Efficacy Stage:**

1

A DECREASE IN RENAL BLOOD FLOW (RBF) is arguably one of the first signs of renal damage in a range of renal disorders.^1^, ^2^ Traditionally, measurement of RBF involves infusion of para‐aminohippurate and blood samples regularly taken over a time‐course of several hours. Clearly, this method is cumbersome and cannot differentiate single kidney blood flow.^3^ These are the main reasons why classical measurement of RBF is hardly used in clinical practice. Phase‐contrast magnetic resonance imaging (PC‐MRI) is a noncontrast‐enhanced MRI technique that allows the determination of blood velocity and flow in a specific vessel during the cardiac cycle within a few minutes.^4^ With no need for contrast agents that are potentially associated with risks for renal patients,^5^ PC‐MRI is a feasible and valid noninvasive technique to reliably measure RBF and a number of derivative hemodynamic parameters, for each kidney separately, in both healthy volunteers and patients with renal or vascular disease.^6^ Moreover, in PC‐MRI RBF is measured directly, in contrast to alternative MRI techniques such as arterial spin labeling (ASL), where total renal perfusion depends on labeling efficiency and the T_1_ of blood and tissue, which are typically estimated. In short, a 3D vascular survey is first acquired to adequately plan the 2D PC‐MRI acquisition plane. Phase and magnitude images are then acquired for each renal artery, and RBF is computed by multiplying the renal artery area by mean blood velocity inside the vessel during the cardiac cycle (Fig. 1),^6^ using the following formula:

$$Q=60\times A\times \bar{v}$$

where Q is the total blood flow rate through the renal artery (in mL/min), 60 is a conversion factor (from seconds to minutes), A is the total vessel area (in cm^2^), and $\bar{v}$ is the average velocity (averaged over the cardiac cycle and vessel area, in cm/s). Renal PC‐MRI has been validated in humans both technically and biologically, also showing good repeatability and reproducibility.^6^, ^7^, ^8^ Despite renal PC‐MRI not being routinely used in the clinic, there are a number of clinical studies showing its potential to support diagnosis and monitoring of renal diseases, in particular chronic kidney disease,^9^ renovascular disease,^10^, ^11^ and autosomal dominant polycystic kidney disease.^12^, ^13^ In healthy subjects, the variability in RBF values across studies is large, likely due to differences in PC‐MRI acquisition and processing, precluding the definition of normative ranges and warranting the need for common protocols facilitating standardization across centers and comparability of different study results.^6^

**FIGURE 1:**
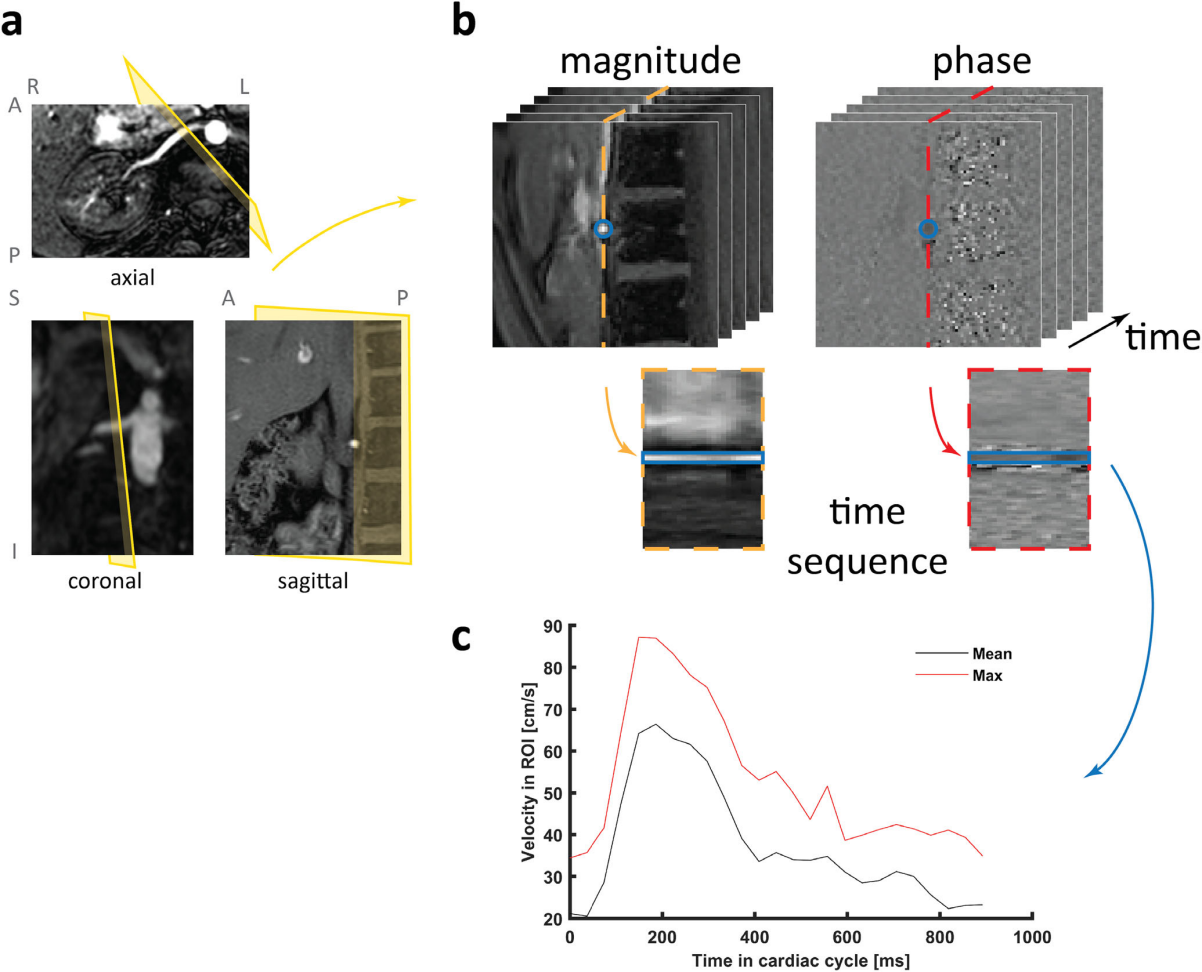
Schematic representation of 2D phase‐contrast magnetic resonance imaging (PC‐MRI) acquisition and processing. (**a**) A single axial, coronal, and sagittal 2D slice of the 3D vascular survey, showing hyperintense arteries, to illustrate planning of the 2D PC‐MRI acquisition plane, (**b**) Acquired phase and magnitude images depicting the renal artery in the center. A circular ROI was drawn on the magnitude images and copied to the phase images. The temporal sequences show the evolution of the phase signal inside the vessel in the cardiac cycle, which is graphically shown in (**c**), a graph showing the mean and max velocity in the ROI during the cardiac cycle. PC‐MRI data were acquired using the recommended acquisition protocol in the University Medical Center Utrecht, the Netherlands and in the ASST Papa Giovanni XXIII, Bergamo, Italy.

The open and growing renal MRI network “Magnetic Resonance Imaging Biomarkers for Chronic Kidney Disease” (PARENCHIMA) is an Action funded by the European Cooperation in Science and Technology (COST, CA16103, www.renalmri.org), which aims at boosting the use of renal MRI biomarkers to improve the management of patients with renal disease. Within this network there is an ongoing effort to improve standardization in the acquisition and analysis of clinical renal MRI data. In line with these aims, the COST Action PARENCHIMA has initiated a consensus project to define expert‐based technical recommendations to harmonize renal MRI acquisition and analysis protocols.^14^ Technical recommendations have been recently published for renal blood oxygenation‐level‐dependent (BOLD),^15^ diffusion‐weighted imaging (DWI),^16^ T_1_ and T_2_ mapping,^17^ and ASL^18^ MRI methods. This study, as part of this standardization effort, proposes a set of technical recommendations for renal 2D PC‐MRI acquisition, analysis, and reporting, based on consensus by an international panel of experts in the field that could establish a clear reference baseline for future developments.

## Materials and Methods

Technical recommendations on subject preparation, scanner hardware, PC‐MRI acquisition, postprocessing, and reporting of results were obtained by consensus, following a previously published consensus defining process.^14^ First, an international panel of experts with first‐hand experience in renal PC‐MRI was formed, as described below. All members were signed up to the panel at their own request, and deliberately consented to participate in the consensus process, which was clearly laid out prior to starting. Then consensus was generated by an approximate Delphi method where, at each iteration, participants were invited to respond to a survey including an anonymous summary of the previous responses.^14^, ^19^, ^20^

### The Panel of Experts

The panel of experts was as large and representative as possible, both in terms of geographical distribution, background, and expertise in order to develop global and comprehensive technical recommendations. The panel included experts with first‐hand experience in renal PC‐MRI as evidenced by a previous track record of publications and/or ongoing research. First, PARENCHIMA members with a publication record and/or research activities in renal PC‐MRI since 2005 were invited to join the expert panel. Then experts participating in similar consensus initiatives on renal DWI,^16^ BOLD,^15^ ASL,^18^ and T_1_ and T_2_ mapping^17^ were contacted and invited if they had research experience in renal PC‐MRI. Last, to further expand the panel of experts, every effort was made to invite at least one researcher or corresponding author from each group contributing to the renal PC‐MRI literature since 2005, as surveyed previously.^6^ When responding to the questionnaires, panelists were asked to list their background/specialty, as well as to specify their expertise in renal PC‐MRI usage. The international panel included 27 experts working in 11 countries from four continents (Europe, America, Asia, and Australia) and with multidisciplinary backgrounds as follows: physics (15/27, 55%), radiology (8/27, 30%), engineering (2/27, 7%), cardiology (1/27, 4%), and mathematics (1/27, 4%). Renal PC‐MRI was mainly used by the panel members for research studies (25/27, 93%), followed by technical development and/or validation (7/27, 26%), and clinical practice (6/27, 22%). The panel members had 6 (interquartile range 4–10) years of experience in 2D PC‐MRI. The panel was formed at the start of the consensus process and remained constant throughout the process. All respondents are listed as authors of this article.

### The Consensus Process

The consensus process took place between January and April 2020. An initial (first round) survey was prepared by the panel co‐chairs (A.d.B. and A.C.) based on the literature and taking advantage of a recently published systematic review and statement paper on renal PC‐MRI.^6^ Information included in published renal PC‐MRI studies was used to identify key preparation, acquisition, and processing protocol parameters. The first‐round survey consisted of 56 questions with multiple‐choice responses, with the possibility to abstain or to provide a long form answer (see Supplementary Material). Participants were prompted to explain the reasoning behind their choices in a comment section following each item and to suggest questions for inclusion in the second‐round survey. The second‐round survey was drafted to include proposed statements informed by the responses and comments from the first round. In the second‐round survey (see Supplementary Material), which consisted of 60 statements, panelists were asked to either agree or disagree with each of the proposed statements; in the case of insufficient experience in any of the statements, panelists were asked to refrain from answering. Both round surveys were administered to the panel members via an online form (Google Forms). Respondents were encouraged to answer the questionnaires based on published evidence and best practices as reflected in the literature, which might deviate from the method they use in their clinical practice.

The consensus process was focused on 2D PC‐MRI. In the first‐round survey there was an additional section on 4D flow (3D Cine PC MR angiography) to determine the amount of expertise on 4D flow in the panel.

With a view to acquiring PC‐MRI as part of a multiparametric renal MRI protocol, subject preparation statements were consistent with those used in previous renal MRI consensus initiatives on BOLD,^15^ DWI,^16^ T_1_ and T_2_ mapping,^17^ and ASL.^18^

### Interpretation of the Survey Results

A “traffic light” system was adopted to issue recommendations based on the degree of consensus achieved by the experts on the individual statements. “Green light” (consensus) was defined as at least 75% unanimity in responses to a question. An “orange light” was defined in the case where the responses showed clear preference (60–74% agreement) without reaching consensus. A “red light” was defined when there was no clear preference by the experts (50–59%); in those cases, no recommendation was possible. For each statement, the percentage of abstentions was also recorded. Agreement and disagreement percentages were computed excluding abstentions. However, the percentage of abstentions for each item was reported, to reflect the level of familiarity of the experts with the topic.

## Results

### Consensus Results

The response rate for both surveys was 100%. The 60 final consensus statements are listed in Table 1. Expertise in 4D flow was limited to 8 out of 27 (29.6%) panel members. Therefore, 4D flow was excluded from further analysis.

**TABLE 1:** Final Consensus Statements on Renal 2D Phase‐Contrast MRI, Formulated by an International Panel of Experts, Following a Modified Delphi Consensus Process

	% Agreement	% Disagreement	% Abstention
**1. Subject preparation**			
1.1 Subjects are required to fast before the scan	38	62	19
1.2 Subject should be scanned in a normal hydration status when clinically appropriate	91	9	12
1.3 Subjects are required to follow a controlled and standardized salt intake before the scan	24	76	35
1.4 Diet should otherwise be controlled (apart from salt and fasting)	0	100	27
1.5 Subjects are not required to fast, but it is recommended to advise them to avoid salty and protein‐rich meals before acquisition	86	14	15
**2. Scanner hardware**			
2.1 2D phase contrast MRI can be performed on both 1.5 and 3T	100	0	8
2.2 The body coil should be used as RF transmitter coil	96	4	0
2.3 A clinical phased array coil should be used as receive coil with the max available receive channels	100	0	4
**3. Preparation of acquisition**			
3.1 B0 shimming is required	79	21	8
3.2 B1 shimming is recommended	68	32	23
3.3 A vascular survey should be performed for planning of the 2D phase contrast MRI	88	12	4
3.4 The vascular survey should be performed at least in coronal and transverse direction to ensure perpendicular planning	100	0	0
3.5 Addition of a sagittal direction to the vascular survey is recommended	64	36	12
3.6 Which vascular survey is used depends on experience and availability in the center, it is suggested to use either IFDIR or TOF MRA in case of a noncontrast MR examination	96	4	8
**4. Planning of 2D phase contrast MRI acquisition**			
4.1 2D phase contrast MRI should be scanned perpendicular to the vessel of interest	96	4	0
4.2 2D phase contrast MRI is preferably planned on the renal arteries	100	0	0
4.3 If planning on the renal arteries is not possible due to limited size or tortuosity of the vessels, it is suggested to measure blood flow through the aorta above and below the branches of the renal arteries	95	5	19
4.4 2D phase contrast MRI should be planned on a linear part of the renal artery without apparent vascular abnormalities (stenoses, string‐of‐beads), preferably not too close to the aorta (roughly >1 cm)	100	0	4
4.5 In case of planning on the aorta, the upper acquisition plane should be placed below the superior mesenteric artery and above the renal arteries.	89	11	31
4.6 In case of planning on the aorta, the lower acquisition plane should be planned below the main renal arteries, below any accessory renal arteries and above the ovarian/testicular arteries	100	0	19
4.7 All renal arteries should be measured independently, including accessory renal arteries. However, if multiple renal arteries happen to run in parallel and perpendicular planning on both is possible, they can be measured in a single acquisition	96	4	0
**5. Acquisition of 2D phase contrast MRI**			
5.1 Fast gradient echo with cartesian readout is currently recommended as a base sequence	100	0	0
5.2 A slice thickness of 3–6 mm is recommended	100	0	4
5.3 The acquired in‐plane voxel size (not the reconstructed voxel size) is recommended to be below 1.5 mm	96	4	0
5.4 The field of view (FOV) should be large enough to avoid foldover artifacts, with the smallest dimension preferably above 200 mm	96	4	0
5.5 The acquired matrix size is related to FOV divided by acquired voxel size. The acquired matrix size is recommended to be larger than 128 × 128	96	4	0
5.6 The shortest possible TE should be used, with a max value of 4 msec	100	0	0
5.7 The shortest possible TR (Siemens and GE: echo spacing) should be used, with a max value of 10 msec	96	4	0
5.8 A flip angle between 10–30 degrees is recommended for noncontrast acquisitions	100	0	0
5.9 Parallel imaging is recommended when there is need to shorten breath‐hold duration	96	4	8
5.10 Halfscan or partial Fourier is not recommended, but if it is required to shorten breath‐hold duration, limited halfscan factors can be used (above 0.7)	100	0	15
5.11 To obtain reasonable SNR, a bandwidth lower than 500 Hz/pixel is recommended	96	4	4
5.12 Fat suppression is not required	91	9	8
**6. Choice of VENC**			
6.1 It is recommended to choose a fixed VENC throughout the study, but check the examination for phase wrapping and repeat with higher VENC if necessary	96	4	0
6.2 For the aorta, a VENC of 150 cm/s is recommended for healthy volunteers	95	5	12
6.3 For the aorta, for populations with suspected vascular disease a VENC of 200 cm/s is recommended	94	6	35
6.4 For the renal arteries, a VENC of 100–120 cm/s is recommended for healthy volunteers	96	4	0
6.5 For populations with suspected vascular disease a higher VENC of 150 cm/s can be indicated	86	14	15
**7. Motion correction**			
7.1 Cardiac synchronization should be performed either using retrospective or prospective triggering	100	0	0
7.2 Cardiac triggering should preferably be performed with ECG	83	17	4
7.3 The number of time points acquired should be maximized within reasonable scan time, with at least 20 time points per cardiac cycle	92	8	0
7.4 Breath‐holding is preferred for respiratory compensation. If impossible, respiratory triggering can be used	91	9	8
7.5 The max breath‐hold time should preferably be below 20s	100	0	0
7.6 If breath‐holding is used, preferably one breath‐hold per artery should be used	100	0	4
**8. Postprocessing**			
8.1 Background phase correction should be performed using stationary voxels during postprocessing if the scanner does not perform it automatically	95	5	23
8.2 Post‐hoc motion correction (ie. image registration, either rigid or affine) is recommended	75	25	19
8.3 A (semi‐)automated approach for ROI selection is recommended, however if that is not available, manual ROI selection can be used	100	0	0
8.4 If ROIs are drawn manually, it is recommended to draw them on each magnitude frame	92	8	4
8.5 In case of manual ROI selection, it is recommended to draw a circular ROI	80	20	0
8.6 In case of artifacts in a single time frame the affected frame should be removed	96	4	0
8.7 In case of artifacts in multiple time frames, the entire examination should be discarded	86	14	12
8.8 Phase unwrapping should be performed if necessary	85	15	23
**9. Reporting of results**			
9.1 It is recommended to report renal blood flow per kidney (so if multiple renal arteries are present, the blood flows through these arteries are combined)	100	0	0
9.2 Average mean velocity is defined as average velocity over time averaged over voxels. It is recommended to report the average mean velocity per vessel	96	4	8
9.3 It is recommended to report the groupwise mean and standard deviation of the average mean velocity per vessel	96	4	8
9.4 The peak systolic velocity is defined as max velocity over time averaged over voxels. It is recommended to report the peak systolic velocity per vessel	83	17	4
9.5 It is recommended to report the groupwise mean and standard deviation of the peak systolic velocity per vessel	83	17	4
9.6 If possible, it is recommended to measure (estimated) GFR, blood pressure and hematocrit as well to be able to calculate filtration fraction, renal vascular resistance and renal plasma flow, respectively	100	0	8
9.7 The parameters listed in Table 3 (left) should at least be reported	≥80^a^	<20	0
9.8 The parameters listed in Table 3 (right) are recommended to be reported as well	≥76^a^	<24	0

Agreement and disagreement percentages were computed excluding abstentions, and were color‐coded as follows: green = consensus (≥75%), orange = preference (≥60%), and red = indeterminate.

^a^ Minimum percentage of agreement for each of the listed items.

The experts showed substantial agreement on renal 2D PC‐MRI data acquisition and analysis protocols: for most of the consensus statements, the percentage of agreement was much higher than the preestablished threshold (≥75%), with 100% agreement in 18/60 (30%) of the statements. Consensus was not reached in 3/60 of the statements, where, however, the experts showed clear preference. Overall, for the 57 statements reaching consensus, the agreement and abstention levels were on average 91.6 ± 9.5% and 7.8 ± 9.4%, respectively.

### Final Recommendations

Considering the literature trends, consensus views, and preferences, the final key recommendations were developed (Table 2).

**TABLE 2:** Key Recommendations on Renal 2D PC‐MRI Acquisition, Processing, and Reporting

Patient preparation	Normal hydration state, avoid salty‐ and protein rich meals
Field strength	1.5 or 3T
Sequence	Fast gradient echo
Vascular survey	Noncontrast‐enhanced MRA
Orientation of imaging plane	Perpendicular to renal artery
In‐plane resolution	<1.5 mm
Slice thickness	3–6 mm
Fat suppression	Not recommended
TR (Siemens and General Electric: echo spacing)	As short as possible, <10 msec
TE	As short as possible, <4 msec
VENC	Healthy volunteers: 100–120 cm/s; Vascular impaired: 150 cm/s
Cardiac synchronization	ECG triggering
Respiratory compensation	Preferably breath‐hold, alternative triggering
ROI placement	Preferably semi‐automated, alternative manual
Reporting	Flow per kidney, mean and peak velocity per vessel
Groupwise reporting	Mean and standard deviation

MRA = magnetic resonance angiography; TR = repetition time (or echo spacing in General Electric and Siemens); TE = echo time; VENC = velocity encoding; ECG = electrocardiogram; ROI = region of interest.

#### RECOMMENDATIONS REGARDING PATIENT PREPARATION

The panel advises against strict control of diet and hydration [Table 1: R1.1–1.5]. Three panelists commented that extensive control of diet and hydration state are hardly feasible in clinical practice. However, subjects should be scanned in normal hydration status [R1.2] and should be asked to avoid salty‐ and protein‐rich meals before acquisition [R1.5], since hydration state, salt, and protein intake are known to influence renal blood flow.^21^, ^22^ It is recommended to measure hematocrit, (estimated) glomerular filtration rage (GFR), and blood pressure since this enables derivation of relevant renal physiology parameters [R9.8]. These include filtration fraction (ratio of the GFR to the renal plasma flow, measuring the proportion of fluid filtered by the kidneys into the renal tubules), renal vascular resistance (ratio of mean arterial pressure to mean RBF, measuring the degree to which the blood vessels of the kidneys impede the flow of blood through them), and renal plasma flow (RPF, blood plasma delivered to the kidneys per unit time, computed as^23^:

$$\mathrm{RPF}=\mathrm{RBF}\;\left(1-hematocrit\right)$$

#### RECOMMENDATIONS REGARDING RENAL 2D PC‐MRI ACQUISITION

**FIGURE 2:**
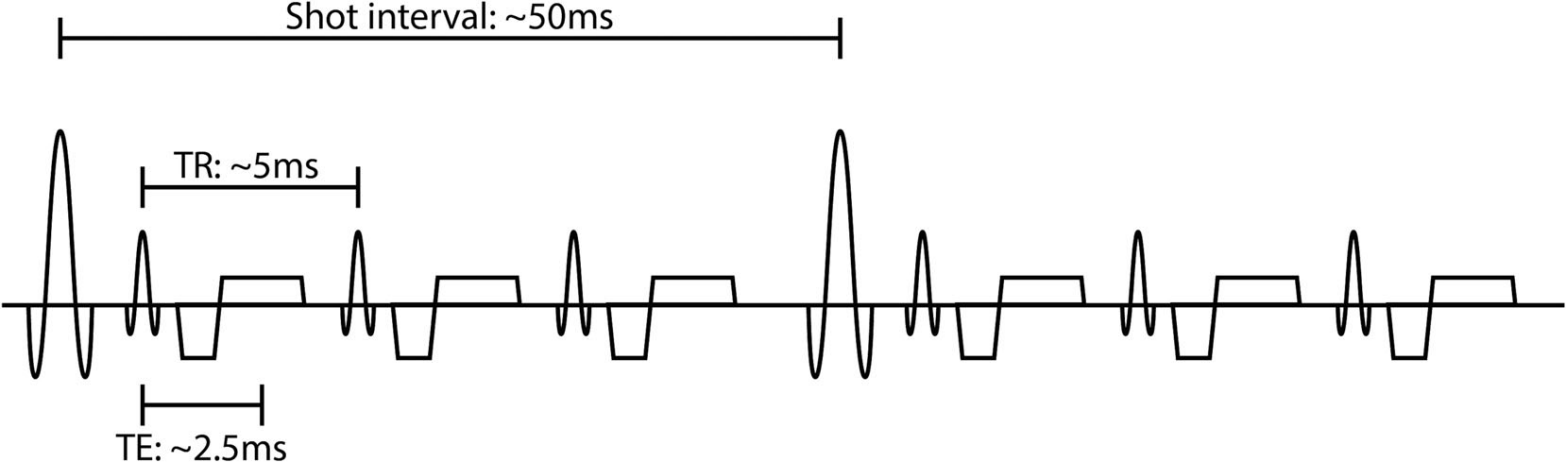
Pulse diagram of the recommended sequence with typical timings of the shot interval (Siemens and General Electric: repetition time), repetition time (Siemens and General Electric: echo spacing), and echo time.

The panel recommends using a fast gradient echo (Philips, Best, Netherlands: T_1_ fast field echo [FFE], Siemens, Erlangen, Germany: fast low angle shot [FLASH]; General Electric, Milwaukee, WI: spoiled gradient recalled [SPGR]) sequence [5.1] with Cartesian readout on a 1.5 or 3T scanner [R2.1]. To minimize scan time, echo time, and repetition time (Philips: repetition time [TR], Siemens and General Electric: echo spacing) should be kept as low as possible (see Fig. 2 for an example pulse sequence diagram) [R5.6–5.7]. For a further reduction of scan time, parallel imaging is preferred over partial Fourier (Philips: halfscan, Siemens: half Fourier; General Electric: fractional number of excitations [NEX]) [R5.9;5.10]. Resolution must be high enough to obtain multiple voxels in the renal artery and low enough to retain a sufficient signal‐to‐noise ratio (SNR), in‐plane resolution below 1.5 [R5.3], and through‐plane resolution of 3–6 mm [R5.2]. Acquisition should be performed in a single breath‐hold, preferably in breath‐out to improve reproducibility, with cardiac triggering using electrocardiogram (ECG) [R7.4–7.6]. In case breath‐holding is impossible (for example, in uncooperative patients), respiratory triggering can be used [R7.4]. Care has to be taken to place the acquisition plane orthogonal to the artery of interest [R4.1]. Therefore, a high‐quality vascular survey in at least two (transverse and coronal) or preferably three orthogonal directions is necessary for planning [R3.3–3.5]. Different noncontrast‐enhanced magnetic resonance angiography (MRA) sequences can provide data of sufficiently high quality, including time‐of‐flight (TOF) MRA and inflow‐dependent inversion recovery (IFDIR)^24^ [R3.6].

Measuring renal blood flow directly on the renal arteries is recommended [R4.2;4.4]. If that is not possible due to tortuosity or small size of the renal arteries, one can instead opt to measure blood flow in the aorta above and below the branching of the renal arteries [R4.3]. In that case, care has to be taken that no other arteries (especially the superior mesenteric artery and the testicular/ovarian arteries) originate between the upper and lower acquisition plane [R4.5–4.6]. Furthermore, all accessory renal arteries should be included [R4.6]. The aortic inflow/outflow method correlates reasonably well with RBF measured directly on the renal arteries.^6^

By default, one acquisition is performed per renal artery with an acquisition (breath‐hold) time below 20 seconds [R7.5–7.6]. Accessory renal arteries should be measured separately, unless multiple arteries happen to run in parallel and can be measured simultaneously [R4.7].

The panel recognizes that there is not a single optimal velocity encoding (VENC) for the acquisition of renal 2D PC‐MRI. In general, a VENC of 100–120 cm/s for the renal artery in healthy volunteers is recommended [R6.4], which should be increased to 150 cm/s in subjects with suspected vascular disease to reflect the higher peak velocities reached in (atherosclerotic) vascular disease [R6.5].^25^ Since peak flow velocity in the aorta is increased,^26^ a higher VENC is recommended for the aorta [R6.2–6.3]. One fixed VENC should be chosen throughout a study; however if phase wrapping is observed, the acquisition should be repeated with a higher VENC [R6.1]. Alternatively, phase unwrapping can be performed (see below) [R8.8].

#### RECOMMENDATIONS REGARDING RENAL 2D PC‐MRI POSTPROCESSING

In addition to the use of breath‐holding during the acquisition, the panel recommends post‐hoc motion correction (ie, image registration, either rigid or affine) [R8.2]. Due to motion of the arteries during the cardiac cycle, images may be misaligned despite the use of breath‐holding. For region of interest (ROI) selection, a semiautomated approach is recommended [R8.3].^6^ The majority of panelists used a threshold‐based approach on either the magnitude (47%) or the phase image (21%), or alternatively a method based on the flow profile (26%). If no software is available for (semi‐)automated processing, it is recommended to draw circular ROIs manually on each magnitude image frame, which eliminates the need for post‐hoc motion correction by image registration, taking care of covering the whole lumen to ensure capturing the whole velocity spectrum [R8.4–8.5]. In case of artifacts affecting a single time frame, that frame should be removed [R8.6]. When artifacts affect multiple time frames, the entire examination should be discarded [R8.7].

#### RECOMMENDATIONS REGARDING REPORTING OF RESULTS

Total renal blood flow per kidney should be reported in milliliters per minute (average renal blood flow over the cardiac cycle). In case of multiple renal arteries, the flow through the main and accessory arteries should be combined [R9.1]. Additionally, it is recommended to report the groupwise mean and standard deviation of the average mean velocity (average velocity over time averaged over voxels) and peak systolic velocity (max velocity over time averaged over voxels) [R9.2–9.5]. In Table 3, all relevant information to report is listed [R9.7–9.8].

**TABLE 3:** Relevant Information to Report in 2D Renal PC‐MRI Studies, Regarding Patient Preparation, Image Acquisition, and Postprocessing

Required	Recommended
Patient preparation (diet, liquid and salt intake)	Artifact handling
Scanner vendor	Background phase correction
Field strength	Matrix size
Base sequence	Partial Fourier/halfscan factor
Geometry (Voxel size, slice thickness, field of view)	Receiver coil
Details on acquisition (TE, TR, flip angle)	Clinical and laboratory parameters – (estimated) GFR, blood pressure, hematocrit
Parallel imaging factor	
VENC	
Details on planning (what vessel, orientation, distance to aorta)	
Details on cardiac synchronization (retrospective/prospective, device)	
Details on respiratory synchronization (breath‐hold/triggering)	
Details on ROI selection (manual/semi‐automated method)	
Scan duration^a^	

^a^ In case of cardiac triggering the actual breath‐hold duration should be reported.

The data in Fig. 1 were acquired using the recommended protocol.

## Discussion

This consensus article provides a set of technical recommendations on renal 2D PC‐MRI acquisition, processing, and reporting to promote standardization across centers, and thus foster the comparability of different study results and the definition of reference values. Furthermore, the current work provides guidelines to new researchers and clinicians approaching renal PC‐MRI for the first time.

### Technical Recommendations

The substantial agreement reached on almost all consensus statements suggests that renal PC‐MRI, being in use in clinical research for more than 20 years, is amenable to standardization and could be sufficiently mature to enter clinical practice.

#### RECOMMENDATIONS REGARDING PATIENT PREPARATION

Renal blood flow has long been known to be influenced by (protein‐rich) meals and hydration state.^21^, ^22^ Postprandially, renal blood flow can be increased by up to 50%,^22^ depending on the composition of the meal. Additional research is needed to determine to what extent control of diet and hydration state is necessary to ensure comparability and repeatability of renal 2D PC‐MRI measurements. Yet practical limitations may lead to difficulties in controlling diet or salt intake.

#### RECOMMENDATIONS REGARDING ACQUISITION

Pragmatically, the panel agreed to recommend different VENC values for the renal artery and the aorta in healthy and vascular‐impaired populations, based on experience and information in the literature on peak velocity in these populations.^25^, ^26^ However, additional research is needed to determine the optimal VENC in different populations. Parallel imaging has been shown not to affect flow estimates and is therefore preferred above partial Fourier.^27^, ^28^

#### RECOMMENDATIONS REGARDING PROCESSING

(Semi‐)automated ROI selection was recommended over manual selection, since it is probably more repeatable, but there was no preference for a specific method. Not all vendors offer semiautomated ROI selection for 2D PC‐MRI and in‐house‐developed tools are currently widely used. There is an urgent need for development of standardized and widely available software and for research comparing (semi‐)automated approaches of ROI selection to manual selection. In Ref. 6 an overview of (semi‐)automated ROI selection methods for renal PC‐MRI is provided.

During postprocessing, background phase correction should be performed as described previously.^29^ On most clinical systems, this is performed automatically by the scanner software.

#### RECOMMENDATIONS REGARDING REPORTING

Currently, the panel recommends reporting RBF in units of ml/min. In case of RBF comparison between groups (rather than to assess changes on individual level), it might be appropriate to correct RBF for body surface area, for example, using the Du Bois formula.^30^

### Issues Reaching Consensus

Experts were divided on the requirement to fast before the scan, as discussed above. Moreover, consensus was not reached on two statements related to preparation of PC‐MRI acquisition. Most experts recommended B_1_ shimming in addition to B_0_ shimming, but the percentage of agreement did not reach the consensus threshold, likely due to limited data available and lack of experience on this issue, proved by a high percentage of abstention. Finally, the experts were divided on the value of performing a sagittal vascular survey in addition to the coronal and transverse vascular survey, with most being in favor. Despite the correct placement of the PC‐MRI acquisition slice determining the accuracy of RBF measurements and derivative parameters, no evidence in the literature about a sagittal vascular survey actually improving PC‐MRI acquisition planning was found. However, a 3D vascular survey can be reformatted by most MR acquisition software in any other plane, so this is a minor issue.

### PC‐MRI as Part of a Renal MRI Protocol

Multiparametric renal MRI protocols are likely to benefit from the combination of PC‐MRI with other promising renal MRI techniques (such as BOLD,^31^ DWI,^32^ T_1_ and T_2_ mapping,^33^ and ASL^34^) providing complementary information on renal microstructure and function in a multiparametric approach,^35^ and thus enabling a complete assessment of the normal and diseased kidney. Considering the acquisition of multiparametric renal MRI data in a single session, patient preparation and scanner hardware guidelines should be consistent across measures.

Our recommendations on scanner hardware are in line with consensus guidelines for renal BOLD,^15^ DWI,^16^ ASL,^18^ and T_1_ and T_2_ mapping,^17^ suggesting that both 1.5T and 3T are adequate field strengths for renal MRI acquisition, despite multiparametric studies being performed more frequently at 3T. Higher field strength provides increased SNR but, conversely, entails greater field inhomogeneities. Moreover, PC‐MRI guidelines recommend a radiofrequency transmitter body coil and a clinical phased array receive coil with the maximum available number of channels, in line with both renal ASL^18^ and T_1_ and T_2_ mapping^17^ guidelines.

Regarding patient preparation for 2D PC‐MRI acquisition, the recommendation to scan subjects in a normal hydration status when clinically appropriate is in line with consensus guidelines for all other renal MRI modalities.^15^, ^16^, ^17^, ^18^ As discussed above for 2D PC‐MRI, also for BOLD, DWI, ASL, and T_1_ and T_2_ mapping evidence on the influence of fasting, diet, and salt intake is currently not sufficient for conclusive statements. This is reflected by the lack of consensus on the requirement to fast in the consensus guidelines for all those renal MRI modalities. More systematic studies are needed to investigate whether fasting, diet, and salt intake control could have an effect on renal multiparametric MRI measurements.

Although acquisition of the 2D PC‐MRI datasets takes two breath‐holds (longer in case of respiratory triggering), planning can be challenging and time‐consuming. A vascular survey is crucial to aid in planning and to assure perpendicular planning of the acquisition plane to the renal arteries. A vascular survey of sufficient quality can be performed within one 20‐second breath‐hold up to 5 minutes (respiratory triggered), depending on the field strength, technical feasibility, and patient cooperation.

### Technical Recommendations, Outlook, and Generalizability

All major vendors of MR systems offer 2D PC‐MRI and the protocol as it is recommended in this article should be feasible on all clinical systems. Nevertheless, the recommendations capture the current consensus of a wide panel of international experts on renal 2D PC‐MRI and are not intended to slow innovation or development in the field in any way. They should not be seen as definitive, but are rather expected and encouraged to be updated over time as more data and technical developments become available. In particular, it is expected: 1) future technical developments to further improve renal PC‐MRI acquisition, for example, flow phantoms as possible methods to calibrate PC sequences^36^ and non‐Cartesian parallel imaging^37^ and compressed sensing^38^ to reduce MRI data acquisition time; 2) increasing expertise in 4D flow, a promising acquisition method allowing extraction of blood velocity and flow information in any plane, rather than in a single double oblique 2D slice,^7^ and increasing evidence that could lead to the development of technical recommendations specific to 4D flow; and 3) improvements in the analysis pipelines, especially in the selection of the ROI and in motion correction. In order to be as generalizable as possible, consensus statements were formulated in vendor‐neutral terms. Future efforts are needed to make vendor‐specific acquisition protocols compliant with the recommendations available through public repositories, to foster their adoption in clinical research, and ultimately in clinical practice. Nevertheless, even without public repositories it should be possible to develop a clinically feasible 2D PC‐MRI protocol for the renal arteries on all clinical systems without the need for any pulse‐programming.

### Limitations

Despite the corresponding authors of all previous renal PC‐MRI articles being contacted and invited to participate in this consensus process, the number of panel members was relatively small. However, the number should be seen in the context of the size of the field. Moreover, the panel included both scientists and clinicians from several international groups that made significant contributions to the field of renal PC‐MRI. Other factors that may have influenced responses, such as age, were not recorded, since the relatively small panel size made it not possible to draw meaningful conclusions from these additional parameters. Lastly, based on their background, renal PC‐MRI type of usage, and specific expertise, the panelists could have insufficient experience to agree or disagree with specific consensus statements. To account for this and avoid biasing the survey, panelists with insufficient experience in any statement were asked to refrain from answering.

### Conclusion

This work provides a set of technical recommendations on renal 2D PC‐MRI acquisition, processing, and reporting, developed by an international panel of experts through a consensus process that should be taken into account when starting new studies in the field of renal PC‐MRI. The current recommendations will promote more widespread adoption of renal PC‐MRI, will foster the initiation of multicenter studies aimed at defining reference values and building larger and more definitive evidence of PC‐MRI clinical validity, and will ultimately allow a PC‐MRI transfer to clinical practice to improve the diagnosis and management of kidney diseases.

## Author Contributions

Conception and design: A.d.B., G.V., C.L., S.S., F.N., and A.C. Acquisition of data: A.d.B., G.V., F.N., and A.C. Data analysis: A.d.B. Interpretation of data: all authors. Drafting of the article: A.d.B., A.C. Critical revision for important intellectual content: all authors.

## Conflicts of Interest

Dr. Ruth P. Lim declares research funding support from Boehringer Ingelheim and See‐Mode Technologies. Dr. Paul D. Hockings is an employee at Antaros Medical. Dr. Michael Bock has Research Cooperation with Siemens Healthcare. Dr. Yasuo Takehara has an endowed chair at Nagoya University, supported by a private company. All other authors have nothing to declare.

## Supporting information


**Appendix S1** Supporting Information.

**Appendix S2** Supporting Information.
