# Supplementary material for: Consensus‐Based Technical Recommendations for Clinical Translation of Renal Phase Contrast MRI
Source: J Magn Reson Imaging. 2020 Nov 2;55(2):323–35. doi: 10.1002/jmri.27419 (PMC9291014; doi:10.1002/jmri.27419)
Supplement: Supplementary file 1 — Appendix S1 Supporting Information. [file JMRI-55-323-s001.pdf]

This questionnaire is part of the COST Action PARENCHIMA (CA16103) initiative working group 1.2 (https://renalmri.org/taskforce/15), which aims to develop technical recommendations for MRI sequences that are short enough to be part of a multiparametric scan with a reasonable scan time.

About this questionnaire:

Any open fields within questions or belonging to questions are only meant for answers to that particular question. If anything is unclear, if you miss something or you would like to add something, please use the "comments" field provided at the end of each section.

In case you don't have enough experience to answer any of the questions, please select the “I have insufficient experience to make a recommendation” option.

Please complete this survey independently if multiple people from your group are participating.

Thank you for your time.

Background of responder

- What is your background?
    - Radiology
    - Physics
    - Nephrology
    - Other, please specify …

Patient preparation

- Subjects are required to fast before the scan
    - Agree, please specify duration in hours
    - Disagree
    - I have insufficient experience to make a recommendation
- Subject should be scanned in a normal hydration status when clinically appropriate
    - Agree
    - Disagree,
    - I have insufficient experience to make a recommendation
- Subjects are required to follow a controlled and standardized salt intake before the scan
    - Agree, please specify in “Other” field
    - Disagree
    - I have insufficient experience to make a recommendation
- Diet should otherwise be controlled (apart from salt and fasting)
    - Agree, please specify in “Other” field
    - Disagree
    - I have insufficient experience to make a recommendation

Acquisition

Please answer the following questions for 2D phase contrast MRI.

Hardware

- Preferred field strength
  - 1.5T
  - 3T
  - Both
  - I have insufficient experience to make a recommendation
- Preferred RF transmit coil
  - Body coil
  - Body array
  - I have insufficient experience to make a recommendation
- Minimum number of receive channels
  - (open)
  - I have insufficient experience to make a recommendation

Scan preparations

- B0 shimming is required
  - Agree
  - Disagree
  - I have insufficient experience to make a recommendation
- B1 shimming is required
  - Agree
  - Disagree
  - I have insufficient experience to make a recommendation
- A vascular survey should be performed for planning of the 2D phase contrast
  - Agree
  - Disagree
  - I have insufficient experience to make a recommendation
- Orientation of vascular survey (tickboxes)
  - Coronal
  - Transversal
  - Sagittal
  - I have insufficient experience to make a recommendation
- What type of vascular survey should be performed?
  - TOF MRA
  - Inflow dependent inversion recovery
  - Other, please specify
  - No recommendation (depending of experience and availability in center)
  - I have insufficient experience to make a recommendation

Planning

- On what vessel is the 2D phase contrast planned?
  - (open)
  - I have insufficient experience to make a recommendation
- In case of planning on the renal arteries, how far from the aorta should the 2D phase contrast be planned?
  - Specify in cm
  - I have insufficient experience to make a recommendation
- In case of planning on the aorta, the upper acquisition plane should be placed below the superior mesenteric artery and above the renal arteries.
  - Agree
  - Disagree
  - I have insufficient experience to make a recommendation
- In case of planning on the aorta, how far from the renal arteries should lower acquisition plane be planned?
  - Specify in cm
  - No recommendation: just stay above any other arteries (like the gonadal arteries)
  - I have insufficient experience to make a recommendation
- In case of planning on the renal arteries, how should the presence of multiple renal arteries be handled?
  - All renal arteries should be measured independently
  - All renal arteries should be measured, but if multiple renal arteries can be measured in a single acquisition, it's OKPerform separate acquisition for each artery
  - Only the main renal artery should be measured
  - I have insufficient experience to make a recommendation

Acquisition parameters

- Which base sequence should be used?
  - Fast gradient echo with cartesian readout
  - Other (please specify)
  - I have insufficient experience to make a recommendation

The following questions are on the geometry parameters of the acquisition. Please provide those values which are in your eyes desirable, yet achievable on most clinical systems. Based on your answers, in the next round of the survey a range of values will be presented and we will ask you whether you consider this range an acceptable recommendation. Which slice thickness should be used? Please specify in mm.

- Which slice thickness should be used? (please specify in mm in the "other" field)
  - (open)
  - I have insufficient experience to make a recommendation
- What voxel size should be acquired? Please specify as …x… in mm.
  - (open)
  - I have insufficient experience to make a recommendation

- What matrix size should be acquired? (please specify as …x… in mm in the "other" field) Matrix size is related to acquired FOV as voxel size x matrix size.
  - (open)
  - I have insufficient experience to make a recommendation
- What TE should be used? Please specify in ms.
  - (open)
  - I have insufficient experience to make a recommendation
- What TR should be used? Please specify in ms.
  - (open)
  - I have insufficient experience to make a recommendation
- What flip angle should be used? Please specify in degree.
  - (open)
  - I have insufficient experience to make a recommendation
- What parallel imaging factor should be used? Please specify.
  - (open)
  - I have insufficient experience to make a recommendation
- What partial fourier or halfscan factor should be used? Please specify (0-1). Specify 1 in case you think partial fourier/halfscan should not be used.
  - (open)
  - I have insufficient experience to make a recommendation
- Which bandwidth should be used?
  - (open)
  - I have insufficient experience to make a recommendation
- 2D phase contrast should be scanned perpendicular to the vessel of interest
  - Agree
  - Disagree
  - I have insufficient experience to make a recommendation
- What fat suppression should be used? If you use a different type, please specify in the free text field.
  - None
  - SPIR
  - SPAIR
  - I have insufficient experience to make a recommendation
- How should the venc be chosen?
  - A fixed venc should be chosen
  - The examination should be checked for phase wrapping, and the examination should be repeated with higher venc if necessary
  - A different venc should be chosen for each patient population, and this venc should be fixed throughout the study
  - I have insufficient experience to make a recommendation

The following questions are on the recommended venc. Please specify in cm/s. If you feel different patient populations need different vencs, please specify this. For example: healthy volunteers: x cm/s; vascular disease: y cm/s.

- What venc would you recommend for the aorta? Please specify in cm/s.
    - (open)
    - I have insufficient experience to make a recommendation
- In case one fixed venc is chosen, what venc would you recommend for the renal arteries?
    - (open)
    - I have insufficient experience to make a recommendation
- In case one fixed venc is chosen, what venc would you recommend for the renal veins?
    - (open)
    - I have insufficient experience to make a recommendation
- Separate acquisitions with toggled gradients should be performed to correct for phase shift due to field inhomogeneities.
    - Agree
    - Disagree, please specify your approach
    - I have insufficient experience to make a recommendation
- Background phase correction should be performed
    - Yes, using a phantom acquisition
    - Yes, using stationary voxels during postprocessing
    - Disagree
    - I have insufficient experience to make a recommendation
- Should cardiac synchronization be performed?
    - Yes, prospective cardiac triggering
    - Yes, retrospective cardiac triggering
    - No, please specify your approach
    - I have insufficient experience to make a recommendation
- What device should be used for cardiac synchronization
    - ECG
    - PPU
    - Either
    - I have insufficient experience to make a recommendation
- If cardiac synchronization is performed, how many time points or cardiac phases should be measured? Please specify.
    - (open)
    - I have insufficient experience to make a recommendation
- If breathholding is used, what is the max acceptable breathhold time? Please specify in seconds.
    - (open)
    - I have insufficient experience to make a recommendation
- If breathholding is used, how many breathholds are acceptable/needed?
    - (open)

- I have insufficient experience to make a recommendation

Postprocessing

- Post-hoc motion correction should be performed
  - Yes, rigid
  - Yes, affine
  - Yes, deformable
  - No
  - I have insufficient experience to make a recommendation
- How should ROI selection be performed?
  - Manual
  - Fully automated
  - Semi-automated
  - I have insufficient experience to make a recommendation
- If automated or semi-automated ROI selection is performed, what kind of technique should be used?
  - Threshold based on the magnitude image
  - Threshold based on the phase image
  - Based on the flow profile
  - Other (please specify)
  - I have insufficient experience to make a recommendation
- If manual ROI selection is performed, on what image should the ROI be drawn?
  - On one magnitude image
  - On one phase image
  - On all magnitude images
  - On all phase images
  - I have insufficient experience to make a recommendation
- If manual ROI selection is performed, what shape should the ROI have?
  - Elliptical
  - Circular
  - Freehand
  - I have insufficient experience to make a recommendation
- How should artifacts affecting the arteries be handled?
  - Removal of the frame with the artifact
  - Discarding the entire examination
  - Other (please specify)
  - I have insufficient experience to make a recommendation
- Phase unwrapping should be performed if necessary.
  - Agree
  - Disagree
  - I have insufficient experience to make a recommendation

Reporting

- What should at least be reported? Please tick boxes.
    - Patient preparation (diet, liquid and salt intake)
    - Scanner vendor
    - Field strength
    - Transmitter coil
    - Receiver coil
    - Base sequence
    - Voxel size
    - FOV
    - Matrix size
    - TE
    - TR
    - Flip angle
    - Parallel imaging factor
    - Partial fourier / halfscan factor
    - Bandwidth
    - B0 shimming
    - B1 shimming
    - Technical details of vascular survey
    - Details on planning (what vessel, orientation, distance to aorta)
    - Venc
    - Use of toggled gradients
    - Details on cardiac synchronization (retrospective/prospective, device)
    - Details on respiratory synchronization (breathhold/triggering)
    - Background phase correction
    - Post hoc motion correction
    - Method of post-hoc motion correction
    - Details on ROI selection (manual/semi-automated)
    - Method of (semi)-automated ROI selection
    - Approach to manual ROI selection
    - Phase unwrapping
    - Artifact handling
    - Other (please specify)
    - I have insufficient experience to make a recommendation
- How should the RBF been reported? Please tick boxes.
    - Total RBF of both kidneys combined
    - Per artery
    - Per kidney (so if multiple renal arteries are present, one value is reported per kidney)
    - I have insufficient experience to make a recommendation
- Flow and velocity should be stated in ml/min and cm/s, respectively.

  - Agree
  - Disagree, please specify
  - I have insufficient experience to make a recommendation
- How should values be reported on a group-wise base? Please tick boxes.
  - Mean blood flow (either per vessel, per kidney or patient as specified earlier)
  - Standard deviation of blood flow (either per vessel, per kidney or patient as specified earlier)
  - Mean max velocity (either per vessel, per kidney or patient as specified earlier)
  - Mean average velocity (either per vessel, per kidney or patient as specified earlier)
  - I have insufficient experience to make a recommendation
- Which other tests are relevant to perform in conjunction with phase contrast MRI? Please tick boxes.
  - Measured or estimated GFR to calculate filtration fraction
  - Hematocrit to calculate renal plasma flow
  - Blood pressure to calculate renal vascular resistance
  - Other, please specify
  - I have insufficient experience to make a recommendation
  - None

4D phase contrast

- Do you have experience in 4D phase contrast imaging of the kidneys?
  - Yes
  - No (the following questions are skipped)
- Please describe the 4D sequence you use?
  - (open)
- Which are, in your opinion, the key aspects to take into account to perform an optimal 4D flow acquisition? Please tick and add anything you consider relevant
  - MRI vendor
  - Image resolution
  - Venc
  - Image orientation
  - Handling of respiratory motion
  - Handling of cardiac motion
  - What to report (flow rate, velocities)
  - Other (please specify)
  - I have insufficient experience to make a recommendation
