# Supplementary material for: Consensus‐Based Technical Recommendations for Clinical Translation of Renal Phase Contrast MRI
Source: J Magn Reson Imaging. 2020 Nov 2;55(2):323–35. doi: 10.1002/jmri.27419 (PMC9291014; doi:10.1002/jmri.27419)
Supplement: Supplementary file 2 — Appendix S2 Supporting Information. [file JMRI-55-323-s002.pdf]

This questionnaire is part of the COST Action PARENCHIMA (CA16103) initiative working group 1.2 (https://renalmri.org/taskforce/15), which aims to develop technical recommendations for MRI sequences that are short enough to be part of a multiparametric scan with a reasonable scan time.

About this questionnaire:

In contrast to the previous questionnaire, the current questionnaire almost exclusively contains statements with "Agree" and "Disagree" options. Those statements have been constructed based on the results of the previous questionnaire.

In case you don't have enough experience to answer any of the questions, please select the "I have insufficient experience to make a recommendation" option.

Please complete this survey independently if multiple people from your group are participating.

Please complete this questionnaire for a non-contrast MR examination, since the COST action is currently focusing mainly on that.

The entire questionnaire focuses on 2D phase contrast MRI. [INCLUDE NOTE FIELD FOR EACH QUESTION ON GOOGLE FORMS!]

## Background of responder

- What is your background?
    - Radiology
    - Physics
    - Nephrology
    - Other, please specify …
- What do you currently use 2D phase contrast for
    - Technical development and/or validation
    - Research studies
    - Clinical practice
    - Other

## Patient preparation

Since the questions regarding patient preparation were standardized in the COST PARENCHIMA recommendation papers, they will not be repeated. Results were:

Consensus: Subject should be scanned in a normal hydration status when clinically appropriate. 95% Agree, 5% Disagree, 4 insufficient experience.

Consensus: Subjects are required to follow a controlled and standardized salt intake before the scan75% Disagree, 25% Agree, 10 insufficient experience.

Consensus: Diet should otherwise be controlled (apart from salt and fasting). 83% Disagree, 11% Agree, 8 insufficient experience.

- Subjects are not required to fast, but it is recommended to advise them to avoid salty and protein-rich meals before acquisition
    - Agree
    - Disagree
    - I have insufficient experience to make a recommendation

## Acquisition

Please answer the following questions for 2D phase contrast MRI as part of a non-contrast MRI examination.

### Hardware

- 2D phase contrast can be performed on both 1.5 and 3T
    - Agree
    - Disagree
    - I have insufficient experience to make a recommendation

The body coil is the coil installed in the magnet bore, in contrast to the local coils placed on the patient.

- The body coil should be used as RF transmitter coil
    - Agree
    - Disagree
    - I have insufficient experience to make a recommendation
- A clinical phased array coil should be used as receive coil with the max available receive channels
    - Agree
    - Disagree
    - I have insufficient experience to make a recommendation

### Scan preparations

Consensus: B0 shimming is required. 83% Agree, 17% Disagree, 3 excluded

Consensus: A vascular survey should be performed for planning of the 2D phase contrast. 88% Agree, 12% Disagree, 1 excluded

Consensus: 2D phase contrast should be scanned perpendicular to the vessel of interest. 100% Agree

- B1 shimming is recommended
    - Agree
    - Disagree
    - I have insufficient experience to make a recommendation
- The vascular survey should be performed at least in coronal and transverse direction to ensure perpendicular planning.
    - Agree
    - Disagree

  - I have insufficient experience to make a recommendation
- Addition of a sagittal direction to the vascular survey is recommended.
  - Agree
  - Disagree
  - I have insufficient experience to make a recommendation

Time of flight (TOF) TOF MRA is defined as a conventional spoiled gradient echo sequence with a somewhat higher flipangle to enhance inflow effects. In Inflow Dependent Inversion Recovery (IFDIR), an extra inversion is added before the readout to null stationary tissue. If used as a vascular survey, (inline) postprocessing is not required.

- Which vascular survey is used depends on experience and availability in the center, we suggest to use either IFDIR or TOF MRA in case of a non-contrast MR examination.
  - Agree
  - Disagree
  - I have insufficient experience to make a recommendation

## Planning

Consensus: In case of planning on the aorta, the upper acquisition plane should be placed below the superior mesenteric artery and above the renal arteries. 89% Agree, 11% disagree, 8 insufficient experience.

- 2D phase contrast is preferably planned on the renal arteries.
  - Agree
  - Disagree
  - I have insufficient experience to make a recommendation
- If planning on the renal arteries is not possible due to limited size or tortuosity of the vessels, we suggest to measure blood flow through the aorta above and below the branches of the renal arteries.
  - Agree
  - Disagree
  - I have insufficient experience to make a recommendation

Stenosis of the renal artery is most often located close to the aorta and might influence the flow profile. (Safian and Textor (2001), doi: 10.1056/NEJM200102083440607)

- 2D phase contrast should be planned on a linear part of the renal artery without apparent vascular anomalies (stenosis, string-of-beads), preferably not too close to the aorta (roughly >1cm).
  - Agree
  - Disagree
  - I have insufficient experience to make a recommendation
- In case of planning on the aorta, the lower acquisition plane should be planned below the main renal arteries and below any accessory renal arteries and above the ovarian/testicular arteries

- Agree
- Disagree
- I have insufficient experience to make a recommendation

- All renal arteries should be measured independently, including accessory renal arteries. However, if multiple renal arteries happen to run in parallel and perpendicular planning on both is possible, they can be measured in a single acquisition.
  - Agree
  - Disagree
  - I have insufficient experience to make a recommendation

## Acquisition parameters

Consensus: Separate acquisitions with toggled gradients should be performed to correct for phase shift due to field inhomogeneities. 86% Agree, 14% disagree, 5 insufficient experience.

- Consensus: Fast gradient echo with cartesian readout is currently recommended as a base sequence.
  - Agree
  - Disagree
  - I have insufficient experience to make a recommendation
- A slice thickness of 3-6 mm is recommended
  - Agree
  - Disagree
  - I have insufficient experience to make a recommendation
- The acquired in-plane voxel size (not reconstructed voxel size) is recommended to be below 1.5 mm
  - Agree
  - Disagree
  - I have insufficient experience to make a recommendation
- The field of view (FOV) should be large enough to avoid foldover artefacts, with the smallest dimension preferably above 200 mm
  - Agree
  - Disagree
  - I have insufficient experience to make a recommendation
- The acquired matrix size is related to FOV divided by acquired voxel size. The acquired matrix size is recommended to be larger than 128x128
  - Agree
  - Disagree
  - I have insufficient experience to make a recommendation

TE and TR parameters tend to differ between vendors for TFE/TurboFLASH/fast GRE sequences. The shot interval is called TR by Siemens and GE. What Philips calls TR is echospacing or ESP for Siemens and GE. The echotime is for all vendors the same. Below a pulse diagram in Philips terminology with some

roughly estimated durations for 2D phase contrast:

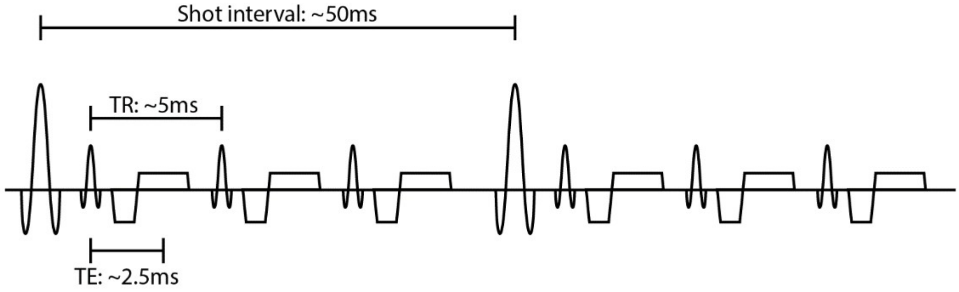


- The shortest possible TE should be used, with a max TE of 4 ms
  - Agree
  - Disagree
  - I have insufficient experience to make a recommendation
- The shortest possible TR (Siemens and GE: echospacing) should be used, with a max TR of 10 ms
  - Agree
  - Disagree
  - I have insufficient experience to make a recommendation
- A flip angle between 10-30 degrees is recommended for noncontrast acquisitions.
  - Agree
  - Disagree
  - I have insufficient experience to make a recommendation

Previous studies showed that parallel imaging does not affect flow estimates by phase contrast MRI. (Bammer R et al. (2007), doi:10.1002/mrm.21109 and Lew CD et al. (2006) doi:10.1016/j.acra.2006.11.008)

- Parallel imaging is recommended when there is need to shorten breath-hold duration.
  - Agree
  - Disagree
  - I have insufficient experience to make a recommendation
- Halfscan or partial Fourier is not recommended, but if it is required to shorten breath-hold duration, limited halfscan factors can be used (above 0.7)
  - Agree
  - Disagree
  - I have insufficient experience to make a recommendation
- Is it possible to use parallel imaging and halfscan in combination at your system? And what system do you use? (open)
- To obtain reasonable SNR, a bandwidth lower than 500 Hz/pixel is recommended
  - Agree
  - Disagree
  - I have insufficient experience to make a recommendation
- Fat suppression is not required.

    - Agree
    - Disagree
    - I have insufficient experience to make a recommendation
- It is recommended to choose a fixed venc throughout the study, but check the examination for phase wrapping and repeat with higher venc if necessary.
    - Agree
    - Disagree
    - I have insufficient experience to make a recommendation

Patients with renal disease often suffer from vascular disease like atherosclerosis as well. Stenosis often leads to higher peak velocities. (Schäberle (2016), doi: 10.1007/s00772-015-0060-3)

- For the aorta, a venc of 150 cm/s is recommended for healthy volunteers.
    - Agree
    - Disagree
    - I have insufficient experience to make a recommendation
- For the aorta, for populations with suspected vascular disease a venc of 200 cm/s is recommended
    - Agree
    - Disagree
    - I have insufficient experience to make a recommendation
- For the renal arteries, a venc of 100-120 cm/s is recommended for healthy volunteers.
    - Agree
    - Disagree
    - I have insufficient experience to make a recommendation
- For populations with suspected vascular disease a higher venc of 150 cm/s can be indicated.
    - Agree
    - Disagree
    - I have insufficient experience to make a recommendation
- Background phase correction should be performed using stationary voxels during postprocessing if the scanner does not perform it automatically
    - Agree
    - Disagree
    - I have insufficient experience to make a recommendation
- Cardiac synchronization should be performed either using retrospective or prospective triggering.
    - Agree
    - Disagree
    - I have insufficient experience to make a recommendation
- Cardiac triggering should preferably be performed with ECG.
    - Agree
    - Disagree

    - I have insufficient experience to make a recommendation
- The number of timepoints acquired should be maximized within reasonable scan time, with at least 20 timepoints per cardiac cycle
    - Agree
    - Disagree
    - I have insufficient experience to make a recommendation
- Breathholding is preferred for respiratory compensation. If impossible, respiratory triggering can be used for respiratory synchronization.
    - Agree
    - Disagree
    - I have insufficient experience to make a recommendation
- The max breathhold time should preferably be below 20s.
    - Agree
    - Disagree
    - I have insufficient experience to make a recommendation
- If breathholding is used, preferably one breathhold per artery should be used.
    - Agree
    - Disagree
    - I have insufficient experience to make a recommendation

## Postprocessing

Consensus: phase unwrapping should be performed. 90% Agree, 10% disagree, 6 insufficient experience

- Post-hoc motion correction (ei. image registration, either rigid, affine) is recommended
    - Agree
    - Disagree
    - I have insufficient experience to make a recommendation
- A (semi-)automated approach for ROI selection is recommended, however if that is not available, manual ROI selection can be used
    - Agree
    - Disagree
    - I have insufficient experience to make a recommendation
- If automated or semi-automated ROI selection is performed, what kind of technique should be used?
    - Threshold based on the magnitude image
    - Threshold based on the phase image
    - Based on the flow profile
    - Other (please specify)
    - I have insufficient experience to make a recommendation
- If you use semi-automated ROI selection, could you please specify which approach you prefer and why? (Open)
- If ROIs are drawn manually, it is recommended to draw them on each magnitude frame

  - Agree
  - Disagree
  - I have insufficient experience to make a recommendation
- In case of manual ROI selection, it is recommended to draw a circular ROI.
  - Agree
  - Disagree
  - I have insufficient experience to make a recommendation
- In case of artefacts in a single time frame the affected frame should be removed
  - Agree
  - Disagree
  - I have insufficient experience to make a recommendation
- In case of artefacts in multiple time frames, the entire examination should be discarded
  - Agree
  - Disagree
  - I have insufficient experience to make a recommendation

## Reporting

Consensus (parameters selected by at least 75%): The following parameters should at least be reported:

1. Patient preparation (diet, liquid and salt intake)
2. Scanner vendor
3. Field strength
4. Base sequence
5. Voxel size
6. TE
7. TR
8. Flip angle
9. Parallel imaging factor
10. Venc
11. Details on planning (what vessel, orientation, distance to aorta)
12. Details on cardiac synchronization (retrospective/prospective, device)
13. Details on respiratory synchronization (breath-hold/triggering)
14. Details on ROI selection (manual/semi-automated)

Consensus: Flow and velocity should be stated in ml/min and cm/s, respectively. 100% Agree

Consensus: The groupwise mean and standard deviation of the blood-flow per kidney should be reported. 100% Agree

- The following parameters should be reported (please tick boxes)
  1. Slice thickness
  2. Method of semi-automated ROI selection (if applicable)
  3. Field of view

4. True scan duration (especially in case of cardiac triggering)

- We recommend to report the following parameters as well (please tick boxes)
    - Artifact handling
    - Background phase correction
    - Matrix size
    - Partial fourier/halfscan factor
    - Receiver coil
- It is recommended to report RBF per kidney (so if multiple renal arteries are present, the blood flows through these arteries are combined)
    - Agree
    - Disagree
    - I have insufficient experience to make a recommendation
- Average mean velocity is defined as average velocity over time averaged over voxels. It is recommended to report the average mean velocity per vessel.Agree
    - Disagree
    - I have insufficient experience to make a recommendation
- It is recommended to report the groupwise mean and standard deviation of the average mean velocity per vessel
    - Agree
    - Disagree
    - I have insufficient experience to make a recommendation
- The peak systolic velocity is defined as max velocity over time averaged over voxels. It is recommended to report the peak systolic velocity per vessel.
    - Agree
    - Disagree
    - I have insufficient experience to make a recommendation
- It is recommended to report the groupwise mean and standard deviation of the peak systolic velocity per vessel
    - Agree
    - Disagree
    - I have insufficient experience to make a recommendation
- If possible, it is recommended to measure (estimated) GFR, blood pressure and hematocrit as well to be able to calculate filtration fraction, renal vascular resistance and renal plasma flow
    - Agree
    - Disagree
    - I have insufficient experience to make a recommendation
